# Supplementary material for: Preparation and characterization of coal-based graphite from Huyan mountain anthracite by high-temperature simulation
Source: PLoS One. 2025 May 2;20(5):e0322558. doi: 10.1371/journal.pone.0322558 (PMC12047810; doi:10.1371/journal.pone.0322558)
Supplement: Attachment — Submitted filename: Response to editor and reviewers.docx [file pone.0322558.s003.docx]

Response to editor

Dear Dr. Anil Kumar Reddy Police:

Thank you for your comments concerning our manuscript entitled “Preparation and characterization of coal-based graphite from Huyan mountain anthracite by high-temperature simulation” (PONE-D-24-53055). Those comments are very helpful for revising and improving our paper, as well as the important guiding significance to our researches. We have studied comments carefully and have made correction which we hope meet with approval. The specific responses are as follows. The responses to the reviewers' comments are placed after the responses to your comments. Revised portion are marked in “Revised Manuscript with Track Changes”.

1. Please ensure that your manuscript meets PLOS ONE's style requirements, including those for file naming.

Response: Thank you for your reminder. During the process of revising, uploading the manuscript and its attachments, all the checks have been carried out as required.

2. In your Methods section, please provide additional information regarding the permits you obtained for the work. Please ensure you have included the full name of the authority that approved the field site access and, if no permits were required, a brief statement explaining why.

Response: Thank you for your suggestion. In the experiments of this research, apart from the high temperature thermal simulation experiment which was carried out at Zhuzhou Nuotian Electrothermal Technology Co., Ltd. (**Line 121 - 123**), the rest of the tests were conducted at College of Geoscience & Surveying Engineering, China University of Mining & Technology, Beijing, China (the 2nd unit of this research), and no relevant approvals or permits were required.

3. Please state what role the funders took in the study.  If the funders had no role, please state: "The funders had no role in study design, data collection and analysis, decision to publish, or preparation of the manuscript."

Response: Thank you for your suggestion. The funders had no role in study design, data collection and analysis, decision to publish, or preparation of the manuscript. Please help supplement it.

4. We note that your Data Availability Statement is currently as follows: [All relevant data are within the manuscript and its Supporting Information files.] Please confirm at this time whether or not your submission contains all raw data required to replicate the results of your study. Authors must share the “minimal data set” for their submission. PLOS defines the minimal data set to consist of the data required to replicate all study findings reported in the article, as well as related metadata and methods.

Response: Thank you for your advice. The data set has been supplemented as per your request and uploaded as an attachment.

In addition, the figures in the manuscript have been proofread in PACE according to your suggestions. Once again, we sincerely thank you for reviewing the manuscript.

Response to reviewers

Dear Reviewers:

We would like to begin with our sincere appreciation for all the valuable comments, insightful suggestions and thoughtful corrections offered by the reviewers to our manuscript “Preparation and characterization of coal-based graphite from Huyan mountain anthracite by high-temperature simulation” (PONE-D-24-53055). The comments and suggestions definitely helped us to improve the quality of the manuscript. We have revised the manuscript in which major changes are highlighted in red in “Revised Manuscript with Track Changes”. These changes are summarized below following a point-by-point response to reviewer's comments.

Reviewer #1

Comment 1: The manuscript is well-organised and contains much informative about the technique and outcomes. Some portions, such as challenges in coal graphitization, it couldn’t explicitly state why overcoming the graphitization threshold is critical and how this research extends existing understanding.

Response: The standard graphite model is established based on Sri Lankan graphite, which is characterized by a carbon layer spacing (*d*_002_) of 0.3354 nm. Prior research has revealed that the closer the *d*_002_ value is to that of standard graphite, the higher the graphitization degree of coal based graphite becomes. Concurrently, significant improvements are detected in its electrical conductivity, initial discharge specific capacity, and thermal conductivity. Notably, coal based graphite manifests a more remarkable electrochemical energy-storage capacity than natural graphite [1]. Furthermore, the coal based graphene synthesized through the Hummers redox method exhibits larger micro-crystalline lamellar structures and fewer defects. These characteristics endow it with distinct advantages in relevant applications. However, the existence of the graphitization threshold impedes the enhancement of the industrial properties of coal based graphite, posing an obstacle to its development and utilization [2]. Therefore, delving into the emergence mechanism of this threshold and exploring effective ways to surmount it are of utmost significance for the advancement of practical applications of coal based graphite. (**Line 452-465**)

[1] Xu X, Cao D, Wei Y, Wang A, Chen G, Wang T, et al. Impact of graphitization degree on the electrochemical and thermal properties of coal. ACS Omega. 2024;9(2):2443-2456. <https://doi.org/10.1021/acsomega.3c06871>

[2] Tang Y, Che Q, Li R, Ma P, Luo P, Ju M, et al. Evolutionary characteristics of coal based graphene structure in West Guizhou and Hanpo’ao,Hunan. Journal of China Coal Society. 2023;48(1):357-372. (in Chinese). [https://doi.org/10.13225/j.cnki.jccs.L022.1275](https://doi.org/10.1021/acsomega.3c06871)

Comment 2: Why was Fe_2_O_3_ specifically chosen as the catalyst in this study? Were other catalysts considered, and how does Fe_2_O_3_ compare in terms of efficiency or relevance to the study's objectives?

Response: Our research team has long been committed to exploring the influence of SiO₂ on the coal graphitization process. As a prevalent mineral in coal, SiO₂ manifests markedly different action mechanisms under high - temperature conditions and high - temperature and high - pressure environments. Leveraging these research achievements, we have published two papers [1-2], which have not only disseminated our findings but also opened up new avenues for further research. Fe₂O₃, another common mineral in natural coal deposits, has been demonstrated by studies conducted by scholars such as Yu [3] and Tang [4] to exert a significant promotional effect on graphitization under high - temperature settings. Inspired by these prior investigations, our research group embarked on a series of experimental endeavors. Specifically, we carried out high - temperature thermal simulation experiments and high - temperature and high - pressure simulation experiments on Fe₂O₃, with the intention of drawing a comprehensive comparison between the two experimental scenarios. Through the high - temperature thermal simulation experiments, we successfully identified the existence of the graphitization threshold, which constitutes the core research content of the present paper. Currently, the body of research on the impact of minerals on graphitization remains relatively limited. In particular, there are notable inadequacies in the systematic analysis of the catalytic effects of diverse minerals. In response to these research gaps, our research group has meticulously devised an experimental plan. This plan involves conducting batch high - temperature graphitization simulation experiments on a range of minerals, including S, F, TiO₂, Fe₃O₄, and Al₂O₃. The overarching objective is to comprehensively summarize the impact of various minerals on graphitization in the future, thereby contributing to the further enrichment and refinement of research in this field.

[1] Chen G, Cao D, Wang A. Wei Y, Liu Z, Zhao M. A high-temperature thermal simulation experiment for coal graphitization with the addition of SiO_2_. Minerals. 2022;12(9):1239. <https://doi.org/10.3390/min12101239>

[2] Chen G, Cao D, Wang A. Wei Y, Liu Z, Zhao M. High-temperature and high-pressure simulation of coal graphitization with SiO_2_ additive. Journal of Mining Science and Technology. 2024;9(2):144-155. (in Chinese) https://doi.org/[10.19606/j.cnki.jmst.2024.02.002](https://dx.doi.org/10.19606/j.cnki.jmst.2024.02.002)

[3] Yu Z, Xie W, Qiu T, Lu Q, Jiang H, He Y. Effect of additives on microstructure of coal-based graphite. Coal Science and Technology. 2023; 51(5):302−308. (in Chinese) https://doi.org/[10.13199/j.cnki.cst.2021-0966](http://dx.doi.org/10.13199/j.cnki.cst.2021-0966)

[4] Tang L, Mao Q, You Z, Yao Z, Zhu X, Zhong Q, et al. Catalytic graphitization in anthracite by reduced iron particles and investigating the mechanism of catalytic transformation via molecular dynamics. Carbon. 2022;188:336-348. https://doi.org/10.1016/j.carbon.2021.12.031

Comment 3: Why is it significant that the Fe_2_O_3_ catalyst facilitates faster defect repair? Explicitly linking these findings to real-world significance would improve the focus.

Response: The phenomenon where the Fe_2_O_3_ catalyst accelerates the defect - repair rate substantiates two key aspects. Firstly, the coal sample from Huyan Mountain has not genuinely attained the upper limit of graphitization and ceased its development process as indicated by the XRD data. In actuality, this data reveals the existence of lattice defects within the Huyan Mountain coal sample, which require further amelioration. This implies that it still harbors substantial development potential and room for improvement. Secondly, this phenomenon comprehensively demonstrates the catalytic effect of Fe_2_O_3_ and lucidly illustrates the specific process and trajectory of its role in promoting graphitization. This finding holds certain reference significance for the subsequent analysis of the mineralization of natural coal based graphite under the influence of minerals, as well as for the simulation research on the graphitization induced by artificial catalysts.

After careful consideration, the fact that Fe_2_O_3_ promotes the healing of in - plane defects is merely a phenomenon observed during the experiments. Additionally, the central focus of this paper lies in the graphitization threshold rather than the catalytic function. Consequently, it is inappropriate to include this as a conclusion. Therefore, the previous conclusion 5 has been expunged and replaced.

Comment 4: The manuscript mentions a fixed coal-to-catalyst ratio (8:2). Was this ratio optimized through preliminary experiments, or was it based on literature? How might varying this ratio affect the results?

Response: Drawing on the high temperature graphitization simulation experiments conducted by Tang [1], in which Fe_2_O_3_ was employed as the catalyst, a proportion of 8:2 was demonstrated to yield highly satisfactory experimental outcomes. Concerning the catalyst proportion, Yu [2], through high temperature thermal simulation experiments, revealed that an increase in the catalyst content generally led to a higher degree of development of the coal based graphite produced. Nevertheless, research by Luo [3] indicated that when the catalyst content surpassed 20%, it had the potential to impede the development of the graphite microcrystalline structure.

Notably, in all previous investigations within our research group on the impact of minerals on graphitization, a ratio of 8:2 was consistently selected, and these endeavors invariably achieved favorable results. In light of the above - mentioned factors, a ratio of 8:2 was determined to be the optimal choice for the present study.

[1] Tang L, Mao Q, You Z, Yao Z, Zhu X, Zhong Q, et al. Catalytic graphitization in anthracite by reduced iron particles and investigating the mechanism of catalytic transformation via molecular dynamics. Carbon. 2022;188:336-348. <https://doi.org/10.1016/j.carbon.2021.12.031>

[2] Yu Z, Xie W, Qiu T, Lu Q, Jiang H, He Y. Effect of additives on microstructure of coal-based graphite. Coal Science and Technology. 2023; 51(5):302−308. (in Chinese) https://doi.org/[10.13199/j.cnki.cst.2021-0966](http://dx.doi.org/10.13199/j.cnki.cst.2021-0966)

[3] Luo P , Tang Y , Li R ,et al.Effects of Minerals Type and Content on the Synthetic Graphitization of Coal: Insights from the Mixture of Minerals and Anthracite with Varied Rank. Minerals, 2023, 13(8). https://doi.org/10.3390/min13081024.

Comment 5: It is stated that the Fe_2_O_3_ catalyst accelerates defect repair and stacking processes but does not alter the graphitization threshold. What is the underlying mechanism limiting further graphitization, and can other methods (e.g., increased pressure or alternative catalysts) potentially overcome this limitation?

Response: Thank you for your suggestion. Based on microscopic analysis and previous studies, we have made speculations on the reasons for the appearance of the graphitization threshold in the discussion. We believe that the main reason for this phenomenon is due to the influence of the graphitization ability of the macerals in coal. （**Line 466-499**）Moreover, existing studies suggest that in order to break the graphitization limitations of macerals, more often than not, it requires the synergistic effect of high temperature and high pressure rather than a single temperature factor. This view is consistent with the formation of coal-measure graphite in nature. Minerals only play an auxiliary role in the graphitization process, and we believe that even with a better catalyst, the threshold will not be broken.

Comment 6: Was there any observable degradation or loss of catalytic activity for Fe_2_O_3_ over the course of the experiment?

Response: Performing detections remains an arduous task. One of the long - standing conundrums that have perpetually confounded our research group is the means of accomplishing real - time observation and recording throughout high - temperature and high - temperature - high - pressure experiments. Over the years, our research group has endeavored to adapt the existing equipment for gas collection and measurement. Nevertheless, the outcomes have fallen far short of expectations. Additionally, operations within high - temperature and high - temperature - high - pressure conditions, such as 3000 °C or 1200 °C - 2 GPa, are rife with peril.

Notwithstanding, upon scrutinizing the evolution curves of parameters including La/Lc/R2/R3 within the temperature span from 2700-3000 °C, a decrease in the graphitization rate compared with 2400-2700 °C was discerned. It is postulated that the principal causes for this decrease are as follows: ① the constraint imposed by the graphitization threshold; ② subsequent to the temperature reaching 2800°C, Fe undergoes vaporization, and a portion of it is expelled from the high - temperature system along with the gas.（**Line 354-357**）

Comment 7: The acid washing and demineralization process is described in detail, but could the presence of residual impurities impact the graphitization threshold? Was this evaluated?

Response: In actuality, subsequent to the demineralization process, XRD testing was performed to validate the efficacy of demineralization. As depicted in Figure 1, apart from the silicon peaks (calibrate with Si), solely the characteristic peaks of carbon (highly metamorphosed anthracite) are discernible, with no conspicuous mineral impurity peaks in evidence.

Figure 1. XRD Pattern of the Demineralized Coal Sample

Comment 8: How does the decrease in interlayer spacing (*d*_002_) relate to potential applications in graphite-based technologies?

Response: Currently, within the identification index system for coal based graphite adopted in China [1], d₀₀₂ serves as a crucial parameter for gauging the degree of graphitization. The closer the value of *d*_002_ approaches 0.3354 nm, the higher the degree of graphitization is manifested. This also implies that the structural characteristics of the experimental sample bear a greater resemblance to those of standard graphite. As stated in Response 1, under such circumstances, the electrical conductivity of the sample is correspondingly more excellent. Once the *d*_002_ value attains a specific standard, this sample can be directly exploited as graphite resources or applied in relevant fields such as graphene preparation.

[1] NB/T 11441-2023, Guidelines for identificationand quality evaluation of coal-measures graphite. China

Comment 9: Figure 1 shows the evolution curve of *d*_002_, but the caption could discuss its implications for achieving the graphitization threshold, there is a lack of clarity.

Response: We sincerely appreciate your suggestions. The captions of numerous figures in the text have been augmented. Specifically, a supplementary description regarding the XRD evolution process of the sample has been incorporated into the caption of Figure 1.（**Line 202-214**）

Comment 10: Could the Fe₂O₃ catalyst's ability to promote graphitization be extended to lower-quality anthracite or other forms of coal like bituminous and low-grade coal? If so, what adjustments to the methodology might be required?

Response: Dear esteemed expert, we are extremely pleased to share with you certain insights concerning the influence of minerals on the graphitization process. Drawing upon prior research works and the investigations carried out by our own team, within the context of high - temperature thermal simulation experiments, when additives such as Fe₂O₃, SiO₂, Ni, and TiO₂ are employed, and provided that an appropriate temperature condition (exceeding 2400 °C) is maintained over a sufficient duration, graphitization can be successfully induced to reach the coal based graphite phase. This outcome holds true regardless of whether the initial coal sample is of low - rank bituminous type or highly metamorphosed anthracite.

Conversely, in the case of using additives such as F and S, a contrary effect is observed. The degree of graphitization of the samples obtained subsequent to the heating process is found to be lower than that of samples without any additives. Evidently, these particular additives exert an inhibitory influence on the graphitization process.

Furthermore, high - temperature and high - pressure graphitization simulation experiments entail a more rigorous experimental setup. Owing to the highly enclosed and high - pressure environment characteristic of such experiments, only coal samples of a rank equal to or higher than lean coal are eligible for experimental use. This is because, during the evolutionary process of lower - ranked coal samples, the gases generated as a result of deoxidation and dehydrogenation reactions cannot be effectively vented, thereby potentially leading to hazardous situations.

In high - temperature and high - pressure experimental scenarios, certain additives, for instance, Fe₂O₃ and Ni, continue to exhibit catalytic properties. This principle equally holds true for the process of synthesizing diamond from graphite under high - temperature and high - pressure conditions, in which ferro - nickel alloy is employed as a catalyst. The underlying reason lies in the fact that both processes essentially entail the evolution of carbon - based structures. However, additives such as SiO₂ display an effect contrary to that observed in high - temperature experiments, with an inhibitory effect being manifested instead.

Reviewer #2

Comment 1: Figure captions are missing.

Response: In accordance with the requirements of PLOS ONE, figures should be uploaded independently. Figure titles and captions are positioned within the main text: Figure 1 (**Line** **202-214**), Figure 2 (**Line** **271 - 272**), Figure 3 (**Line 298 - 303**), Figure 4 (**Line** **358 - 359**), Figure 5 (**Line 380 - 383**), Figure 6 (**Line 411 - 423**), Figure 7 (**Line 442 - 443**), Figure 8 (**Line 500 - 501**). Moreover, during the revision, detailed content descriptions were incorporated into multiple figure captions (Figure 1, 3, 5, 6).

Comment 2. XPS data needs to be added.

Response: Thank you for your suggestion. The essence of graphitization resides in a process whereby, concomitant with the escalation of the degree of coal metamorphism, the large - molecular structural units (BSUs) of coal undergo the cleavage of a profusion of bridge bonds (-CH₂-, -O-CH₂-), and elements extraneous to carbon are sequentially expelled. Oberlin [1] segregated graphitization into two constituents. Initially, a six - membered aromatic carbon structure is engendered, and subsequently, through the polymerization and rearrangement of the aromatic ring structure, a planar carbon layer is fashioned. In essence, it represents a process of deoxygenation, dehydrogenation, and the rupture of bridge bonds to erect a three - dimensional ordered graphite structure.

In the antecedent research on coal based graphite, it has emerged as a consensus among scholars to conduct inquiries using methodologies such as XRD, Raman, SEM, and HRTEM. Conversely, the application of XPS has been comparatively circumscribed. Merely a handful of scholars have employed it to ascertain the elemental composition and functional groups during the developmental progression from highly metamorphosed anthracite to coal - based graphite. In the current study, the samples subjected to high - temperature treatment have all achieved a relatively high graphitization degree. As discerned by transmission electron microscopy, a relatively complete three - dimensional graphite structure has been formed internally. The interior is preponderantly composed of the covalent bonds of graphite, with a high - grade enrichment of carbon elements, and most other elements have been expunged. Consequently, we postulate that conducting XPS tests in this study will not yield propitious results and is not a necessary experiment. Herein, we proffer an application to you asserting that there is no exigency to supplement XPS tests.

[1] Oberlin A. High resolution TEM studies of carbonization and graphitization. In: Thrower PA, editor. Chemistry & physics of carbon. 1st ed. Boca Raton: CRC Press; 1989.

Comment 3. Element compositions (C, H, N, and S) of the coal-based graphite need to be mentioned.

Response: Thank you for your invaluable suggestions. In actuality, we have carried out elemental analysis on the post - experimental coal based graphite. As elucidated in Response 1, at the stage of highly metamorphosed coal based graphite, the content of elements other than carbon has declined to an infinitesimal value. The variation trend of elements within the temperature range of 2100 - 3000 °C is extraordinarily tenuous and bereft of a conspicuous pattern. Furthermore, as illustrated by Zhang's [1] investigation, through continuous thermal ramping, it was determined that the majority of elemental content changes predominantly occurred during the heating period from ambient temperature to 1000 °C. In the 1000 - 3000 °C regime, the change in content was minuscule, and the evolution curve approximated a horizontal line (Figure 1), which bears a striking resemblance to the experimental phenomena in our study. Therefore, after comprehensive deliberation, we have resolved not to incorporate the element content changes with nebulous patterns and minimal analytical significance into the article, so as to preclude any potential confusion among experts and readers. We earnestly hope for your understanding.

Figure 1.Componential variations samples with high temperature treatments (Zhang, et al. 2023)

[1] Zhang X, Wang S, Chen H, Wang X, Deng J, Li X, et al. Observation of carbon nanostructure and evolution of chemical structure from coal to graphite by high temperature treatment, using componential determination, X-ray diffraction and high-resolution transmission electron microscope. Fuel. 2023;332(Pt 1):126145. https://doi.org/10.1016/j.fuel.2022.126145

Comment 4. FTIR and SEM need to be carried out.

Response: Appreciate your counsel. The scanning electron microscopy (SEM) examination of the post - experiment samples has been duly executed as per the requirements, and the pertinent analysis has been performed (**Line 192 - 195, 431 - 442**). However, the FTIR test holds limited utility within the context of this study. Even the FTIR spectra obtained at the highly metamorphosed coal stage are notably feeble [1]. A prior investigation by Guo et al. [2] revealed that upon the pyrolysis temperature escalating to 950 °C, the heat treatment disrupted the chemical structure of coal and eradicated the structural disparities among coals of diverse ranks. The research by Li [3] demonstrated that at 500 °C, functional moieties such as methyl, methylene, and aromatic rings within the raw anthracite coal remained preserved. When the heating temperature reached ≥ 1000 °C, nearly all C - H bonds vanished. Given the ultra - high temperature conditions employed in this experiment, the fabricated samples have attained a substantial degree of graphitization. Consequently, following meticulous analysis, it is opined that performing the FTIR test is unlikely to yield favorable outcomes and will not contribute significantly to the study.Your comprehension is sincerely solicited.

[1] Li, K. Investigation on the Structural Ordering of Natural Coaly Graphite from Xinhua, Hunan Province, China. China University of Mining＆Technology-Beijing, Beijing, China. (Doctoral dissertation in Chinese)

[2] Li, K.; Zhu, Y.; Cao, H.; Zhang, H.; Wu, Y.; Li, X.; Xu, Z.; Liu, Q. Graphite Made from Coal by High-Temperature Treatment: An Insight into the Nanometric Carbon Structural Evolution. Minerals 2024, 14, 1092. https://doi.org/10.3390/ min14111092

[3] Guo, X.; Tang, Y.; Schobert, H.H.; Eble, C.F.; Chen, C. Inspired by the Optical Properties of Char and Coke: A Study on Differences between Them from Perspectives of Organic Elemental Contents and the Carbon Nanostructure. Energy Fuels 2024, 38, 3713–3727.

Reviewer #3

Comment 1: Include the discussion about the role of catalyst (Fe_2_O_3_) and compare with the other available catalysts and conditions reported in literature.

Response: Thank you for your insightful remarks. The mechanism by which Fe₂O₃ exerts an influence on graphitization has been appropriately integrated into the main text (**Line 109 - 113**). Notably, the current paper is principally concerned with the graphitization process of anthracite and the determination of the graphitization threshold within this process. It does not principally concentrate on the aptitude of additives to affect graphitization. Moreover, given that the coal rank, provenance, composition of coal samples, and experimental conditions in the extant research by scholars exhibit substantial divergence and lack standardization, rendering direct comparison impracticable, we earnestly solicit your comprehension.

Comment 2. All the spectra given in the Figure 3 are very low resolution and hard to read or analysis.

Response: Thank you for bringing this to our attention. In accordance with the requirements of the PLOS ONE journal, the figures in the manuscript have been re-uploaded after undergoing verification in PACE. For the pictures that are difficult to see clearly, it may be because they were compressed when the website generated the PDF. Please click on the link in the upper right corner to download the original images.

Comment 3. Very low resolution images are used in other figure also. Give high resolution images (Fig 5, Fig 6).

Response: Similar to the Response 2, the figures have been verified and then re-uploaded.

Comment 4. Novelty of this work is poorly described.

Response: Dear Esteemed Expert, We are profoundly obliged to you for your invaluable and incisive observations. The concern regarding the dearth of innovation that you propounded is of paramount significance to us. Herein, we intend to proffer a comprehensive and meticulous explanation and elucidation on this particular facet.

In the domain of research content, despite the extensive and exhaustive prior investigations into the graphitization of coal, our study presents a distinct and novel perspective. We concentrate our focus on the highly metamorphosed anthracite from Huyan Mountain in Shanxi Province. Its elevated state of metamorphism furnishes a novel and unique sample substrate for our research undertakings. Through experimental exertions, we have unearthed a well - defined and unambiguous graphitization threshold (*d*_002_ = 0.3368nm) for this anthracite. This discovery constitutes a substantial and momentous augmentation to the theoretical edifice of coal graphitization. Although previous studies have delved into the coal graphitization process and some scholars have obliquely detected the existence of a graphitization threshold, none have conducted elaborate and meticulous testing and analysis centered around this phenomenon. Our discovery facilitates a more profound and in - depth comprehension of the underlying mechanisms of coal graphitization and demarcates the pivotal junctures within the graphitization process.

With regard to the experimental methodology, considering the temperature constraints imposed by the instrumentation, we have ingeniously and resourcefully adopted the approach of elevating the initial metamorphic degree of the coal sample and introducing a catalyst to redress the deficiency in temperature conditions. This enables the coal sample to attain a higher echelon of graphitization within a circumscribed temperature range. This experimental design not only proffers a novel and innovative concept for researching coal graphitization under high - temperature scenarios but also serves as a paradigmatic exemplar for formulating experimental protocols in subsequent related research.

Moreover, this study systematically amalgamates an array of characterization techniques, including XRD, Raman spectroscopy, SEM, and HRTEM, to comprehensively and penetratingly analyze the microstructural evolution, crystal lattice construction, and defect rectification during the graphitization process. This integrative and comprehensive analytical approach, which harnesses multiple techniques, furnishes robust and steadfast support for precisely unravelling the coal graphitization process and also evinces a certain degree of innovation within analogous research undertakings.

Furthermore, during the revision phase, we have concisely expounded upon the implications of the graphitization threshold for the practical exploitation and application of coal - series graphite, in light of the prevailing circumstances. Additionally, grounded in microscopic observations and prior research findings regarding the graphitization of microscopic components in coal, we have deliberated on the genesis of the graphitization threshold, thereby laying the groundwork for further in - depth analysis.(**Line 466 - 499** )

We are acutely cognizant that research work necessitates continuous refinement and profundity. Your insights are pivotal and instrumental for us to further enhance the caliber and quality of our research. Once again, we extend our gratitude for your attention and support. We eagerly anticipate receiving more of your sagacious guidance in our subsequent research pursuits.

Comment 5. The manuscript needs improvement scientifically.

Response: Dear Esteemed Expert, regarding your perspective that the article lacks scientific rigor, following meticulous deliberation and organization, we firmly assert that the article adheres to high scientific standards throughout the research process. The justifications are presented as follows:

① The experimental design is both rigorous and rational. In the aspect of sample selection, we opted for the highly metamorphosed anthracite from Huyan Mountain in Shanxi Province as the raw material. Its maximum vitrinite reflectance (Ro, max/%) attains 6.32, categorizing it as a coal sample with a high metamorphic degree. This selection is grounded in prior research, considering that the high - temperature graphitization processes of coal samples with diverse metamorphic degrees diverge significantly. A highly metamorphosed coal sample can enter the graphitization stage earlier during the heating process. This enables the experiment to concentrate on the more research - significant aspects and effectively regulate the impact of initial conditions on the experimental outcomes. In the experiment, an experimental group with the addition of Fe₂O₃ catalyst and a control group without such addition were established. The contrast is distinct. That is, through this comparison, the existence of the graphitization threshold was validated, and it also facilitates the exploration of the catalyst's influence on the anthracite graphitization process. This control - experiment design, a prevalent approach in scientific research, bolsters the persuasiveness of the experimental results.

② The research methodology is scientific and dependable. In the high - temperature simulation experiment, the NTG - SML - 60W integrated laboratory graphitization furnace manufactured by Zhuzhou Nuotian Electric Heating Technology Co., Ltd. was employed, and the segmented heating method was utilized while strictly controlling the experimental conditions. For instance, prior to heating, the gas was evacuated to 5 Pa and replaced once, and argon gas with a flow rate of 10 L/min was introduced throughout the experiment for protection. The heating rate, constant - temperature duration, and target - temperature settings are explicit and rational. These operations conform to the norms of high - temperature experiments, ensuring the stability and reproducibility of the experimental conditions. Multiple analytical techniques, such as X - ray diffraction (XRD), laser Raman spectroscopy, high - resolution transmission electron microscopy (HRTEM), and scanning electron microscopy (SEM), were applied to characterize the samples. These methods are extensively used in the field of coal based graphite analysis. By analyzing the microstructure, lattice defects, and graphitization degree of the samples from multiple perspectives via different methods, they corroborate one another, guaranteeing the scientific nature of the research findings. For example, XRD is used to compute the lattice parameters (the interlayer spacing of carbon layers d_002_, the extension La, and the stacking Lc), Raman spectroscopy is used to analyze the structural defects, and HRTEM and SEM are used to visually observe the construction process of the graphite lattice. The combination of multiple methods comprehensively and profoundly reveals the characteristics of anthracite graphitization.

③ The result analysis is objective and comprehensive. For the analysis of the experimental results, we carried out a systematic and in - depth discussion based on the data acquired from multiple characterization methods. For example, through the analysis of XRD data, we ascertained that there is a graphitization threshold of d_002_ = 0.3368 nm for the Huyanshan anthracite. We elaborated in detail on the variation patterns of parameters such as the interlayer spacing of carbon layers, the extension of aromatic lamellae, and the stacking degree within different temperature ranges, as well as the influence of the catalyst on these parameters. In the Raman spectroscopy analysis, parameters like R2 and R3 were used to characterize the structural defects of the samples, clearly presenting the process of lattice defect repair and graphite lattice construction within the samples as the temperature rises, further verifying the existence of the graphitization threshold and the mechanism of the catalyst's action. The observation results of HRTEM are in consonance with the XRD and Raman spectroscopy analyses, visually demonstrating the development of carbon layers and the evolution of the lattice within the samples at different temperatures, providing robust support for the conclusions at the micro - level.

In summary, the article adheres to the norms and principles of scientific research in experimental design, research methodology, and result analysis, demonstrating strong scientific rigor. Of course, we are well - aware that scientific research is an ongoing process of refinement. Should you have any further specific comments or suggestions, please feel free to point them out. We will deliberate on them carefully and make positive improvements. Thank you again for your attention and guidance!
